# Relationship of physical activity and cognitive functioning among breast cancer survivors: a cross-sectional analysis

**DOI:** 10.3389/fcogn.2024.1332960

**Published:** 2024-05-09

**Authors:** Sheri J. Hartman, Rong W. Zablocki, Rowena M. Tam, Barton W. Palmer, Barbara A. Parker, Dorothy D. Sears, Tim A. Ahles, Loki Natarajan

**Affiliations:** 1 Herbert Wertheim School of Public Health, UC San Diego, La Jolla, CA, United States; 2 UC San Diego Moores Cancer Center, UC San Diego, La Jolla, CA, United States; 3 Department of Psychiatry, UC San Diego, La Jolla, CA, United States; 4 Veterans Affairs San Diego Healthcare System, San Diego, CA, United States; 5 Department of Medicine, UC San Diego, La Jolla, CA, United States; 6 College of Health Solutions, Arizona State University, Phoenix, AZ, United States; 7 Department of Family Medicine, UC San Diego, La Jolla, CA, United States; 8 Department of Psychiatry and Behavioral Sciences, Memorial Sloan Kettering Cancer Center, New York, NY, United States

**Keywords:** survivorship, exercise, aerobic activity, cognition, CRCI, objective physical activity

## Abstract

**Introduction:**

Cancer related cognitive decline is a common long-term side effect of cancer and its treatments among breast cancer survivors. Physical activity is a modifiable risk factor related to cognitive decline. However, existing research lacks consensus regarding the relationship between cognition and exercise as well as the impact of cancer treatments on this relationship. Baseline data from an ongoing randomized clinical trial was utilized to examine the relationship between self-reported and objectively measured cognition with physical activity. Exploratory analyses examined cancer treatments as potential moderators.

**Methods:**

Breast cancer survivors (*N* = 253) completed a battery of neurocognitive tests, the PROMIS Cognitive abilities questionnaire, medical charts abstracted for treatment information, and wore an ActiGraph accelerometer at the waist for 7 days. Data were analyzed using multiple linear regression models.

**Results:**

Participants were on average 58.5 (SD = 8.88) years old, diagnosed 3 years prior to enrollment (SD = 1.27) with 57% treated with chemotherapy and 80% receiving hormone therapy at baseline. Better self-reported cognitive ability was significantly associated with greater min of moderate to vigorous physical activity (MVPA; β = 0.070, se = 0.028, *p* = 0.012). There were no significant associations with any objectively measured cognitive domains. Time since diagnosis (years) was a significant moderator of MVPA and Processing Speed (β = −0.103, se = 0.043, *p* = 0.017). Treatment with chemotherapy and/or hormones did not significantly moderate the relationship between MVPA and any of the cognitive measures or domains.

**Conclusion:**

Findings suggest that physical activity is related to self-reported cognition but not objectively measured cognition. Greater physical activity was associated with faster processing speed in participants closer in time to their cancer diagnosis. These results emphasize the need for more research to understand when cancer survivors may benefit from physical activity and what aspects of cognition may be improved.

## Introduction

Breast cancer is the most common cancer diagnosis among women (American Cancer Society, 2018) with more than 4 million survivors in the US (Miller et al., 2022). While chemotherapy and endocrine therapy have improved disease-free and overall survival for breast cancer survivors, a common and often long-term negative consequence of these treatments is problems with cognition (Kohli et al., 2007; Janelsins et al., 2014, 2017; Bernstein et al., 2017; Rosenfeld et al., 2018; Gervais et al., 2019; Haggstrom et al., 2022). Overall, one in three breast cancer survivors experiences cognitive cancer-related cognitive decline (Whittaker et al., 2022) (CRCD) which includes difficulties with attention, memory, executive function, and processing speed (Anderson-Hanley et al., 2003; Falleti et al., 2005; Jansen et al., 2005; Jim et al., 2012; Janelsins et al., 2014; Ono et al., 2015; Hardy et al., 2018). CRCD can also negatively impact quality of life, daily functioning, and ability to return to work (Steiner et al., 2004; Wefel et al., 2004; Lauzier et al., 2008; Reid-Arndt et al., 2009; Duijts et al., 2014; Jagsi et al., 2014). Among women who were treated with chemotherapy, on average 44% self-report cognitive difficulties and 21%–34% display impairments on objective tests of cognition (Whittaker et al., 2022). Studies examining breast cancer survivors who have undergone endocrine treatment report similar findings with worse processing speed (Collins et al., 2009; Chen et al., 2014), memory (Shilling et al., 2003; Palmer et al., 2008; Schilder et al., 2009) and overall cognitive function (Phillips et al., 2010). While both chemotherapy and endocrine therapy have individual impacts on cognitive function, a recent prospective study in breast cancer survivors found no incremental deterioration in the presence of both therapies (Mandelblatt et al., 2016). A growing research literature demonstrates the benefit of physical activity for CRCD, however some results have been discrepant in part due to variations in how both cognition and activity are assessed (Brunet and Sharma, 2023).

The existing research on the relationship between cognition and exercise for breast cancer survivors has lacked consensus, the strongest evidence remains with the relationship between self-reported cognition and processing speed (Marinac et al., 2015; Campbell et al., 2019; Brunet and Sharma, 2023). Although self-report and objective measures of cognition are often not related, both measures capture important aspects of cancer survivors experience (Tannock et al., 2004; Bender et al., 2008; Hutchinson et al., 2012). For objective cognitive domains, processing speed is commonly impacted by chemotherapy and endocrine therapy (Wefel et al., 2004; Ball et al., 2007; Correa and Ahles, 2008; Deary et al., 2009; Ahles et al., 2010; Haggstrom et al., 2022). Information processing speed is the ability to take in information and use it quickly and appropriately and is central to overall cognition and can impact memory and ability to learn new tasks (Salthouse, 1996; Ball et al., 2007).

Understanding what aspects of cognition are related to activity, and when and who would most benefit from physical activity is still unknown (Meattini et al., 2017; Campbell et al., 2019; Erlenbach et al., 2021; Haggstrom et al., 2022; Brunet and Sharma, 2023; Franco-Rocha et al., 2023; Jesús et al., 2023). Therefore, the primary aim of the current analyses was to test the relationship between self-reported cognitive abilities and objectively measured processing speed with accelerometer measured moderate to vigorous physical activity (MVPA). Our a priori hypothesis was that greater minutes of daily MVPA would be associated with self-reported cognition and objectively measured processing speed. Our secondary aim, was to explore the relationship of minutes of MVPA with three objectively measured cognitive domains: memory, executive function, and attention. We hypothesized that greater minutes of daily MVPA would be associated with better domain scores for all three domains. Lastly, we examined whether time since diagnosis (years) and treatment type (chemotherapy and/or hormone therapy) moderated any of the relationships between self-reported and objective cognition and physical activity.

## Methods

### Participants

Baseline data from breast cancer survivors who were enrolled in an ongoing 12-month physical activity randomized controlled trial were used for the current analyses. The study protocol was published previously (Hartman et al., 2021). Briefly, inclusion criteria were female breast cancer survivors diagnosed with stages 1–3 breast cancer within the prior 5 years, at least 40 years of age, completed active treatment (e.g., chemotherapy, radiotherapy) at least 6 months prior to enrollment, received endocrine therapy and/or chemotherapy as part of their treatment, self-reported engagement in <60 min per week of MVPA accumulated in 10-min bouts, self-reported difficulties with cognition with a score of 4 or higher on a 0–10 scale (Williams et al., 2018) and possession of a Fitbit compatible device (e.g., smartphone, computer) with Internet access. Exclusion criteria included any medical condition that could make it unsafe to participate in unsupervised physical activity, currently taking tamoxifen or an aromatase inhibitor that was planned for discontinuation in the subsequent 6 months, and inability to commit to a 12-month study. Baseline data, from the complete sample of enrolled participants were collected from September 2019 to December 2022 and analyzed June-October 2023. The UC San Diego institutional review board approved all study procedures and all participants provided written informed consent.

The primary recruitment method was through registry lists of breast cancer survivors from the California Cancer Registry and the UC San Diego Epic electronic medical record (with IRB approved HIPAA-waiver for medical record screening). Potential participants were mailed a letter and flier about the *I Can!* Study that included instructions on how to opt out of subsequent contact or contact the study to learn more. Potential participants who did not opt out were contacted by email, text message, or phone call from the study staff up to 3 times. Potential participants were screened for eligibility over the phone with interested and eligible participants scheduled for an in-person orientation/measurement visit. At the in-person visit, participants signed informed consent, completed baseline measures (see Measures section), and were fitted with a hip worn accelerometer, an ActiGraph GT3X+ (ActiGraph, LLC) to wear during waking hours for the next 7 days. Participants returned to the clinic after at least 7 days, the ActiGraph was screened for adequate wear time, and any remaining baseline measures were completed. Participants receive $20 for completing the baseline measures.

### Measures

The trial has two primary cognitive outcomes—one self-reported and one objectively measured. The primary self-reported cognition measure was the computer adaptive testing form of the Patient Reported Outcomes Measurement Information System (PROMIS) Cognitive Abilities scale. This scale assess individuals’ perceptions of their cognitive abilities in the areas of mental acuity, concentration, verbal and nonverbal memory, and verbal fluency, as well as perceived changes in these cognitive functions (Lai et al., 2014).

The primary objective cognitive outcome was Processing Speed, as measured with the Oral Symbol Digit test from the NIH Toolbox Cognition Domain (nihtoolbox.org) (Heaton et al., 2014; Weintraub et al., 2014). The measure is a computer-based analog to the Wechsler Adult Intelligence Scale Digit-Symbol-Coding test and has been validated and normed in individuals aged 3 to 85 years.

Secondary cognitive domains were objectively measured Memory, Executive Function, and Attention. Paper-and-pencil tests were selected from the consensus battery of International Cognitive and Cancer Taskforce (ICCT) (Wefel et al., 2011) and supplemented with computerized measures from the NIH Toolbox Cognitive Domain and the Conners’ Continuous Performance Test 3 (CPT-3). The Memory Domain was assessed with the NIH Toolbox (Bender et al., 2024) Picture Sequence Memory and List Sorting Working Memory Tests, and 2 scores from the Hopkins Verbal Learning Test-Revised (Benedict et al., 1998) (HVLT-R): summary of the 3 immediate recall scores and the delayed recall score. The Executive function Domain was assessed with time to complete Trail Making Test–Trails B (Reitan, 1958) and the NIH Toolbox Dimensional Change Card Sort Test (Bender et al., 2024). The Attention Domain was assessed with the NIH Toolbox Flanker Inhibitory Control and Attention Test (Bender et al., 2024) and with four subscales of the CPT-3: Detectability (d’), Variability, Hit Reaction Time Block Change, Hit Reaction Time Inter-Stimulus Intervals Change (Conners et al., 2000). National Adult Reading Test-Revised (NART-R) (Nelson and Willison, 1991) was administered as a measure of crystalized verbal knowledge as a means to adjust for estimated premorbid cognitive function in data analyses.

An overall z-score was derived for each domain for the secondary cognitive outcomes (Memory, Executive function, and Attention), by first creating a z-score for each individual test score and then averaging the z-scores for each domain. Any component in a domain that was not age-corrected, was residualized by age first, and the residual was standardized to z-score (i.e., we regressed the cognitive outcome on age and extracted the residuals). In order to ensure that higher domain score indicated better cognitive function, for any component that has a negative relationship with cognitive function, the z-score was multiplied by −1 before averaging to the final domain score.

Physical activity intensity assessed with the ActiGraph GT3X+ worn at the waist. The ActiGraph GT3X+ provides second-by-second estimates of activity that can be categorized into minutes spent in MVPA using determined based on the validated Freedson cut point of 1952 or higher (Freedson et al., 1998). Its ability to measure physical activity with fidelity has been validated against heart rate telemetry and total energy expenditure (Melanson Jr and Freedson, 1995; Plasqui and Westerterp, 2007). Sufficient wear time was defined as 5 days with ≥600 min of wear time or 3,000 min (50 h) across 4 days; anyone with less than the minimum wear time was asked to re-wear the ActiGraph.

Breast cancer diagnosis and treatment was collected via medical chart reviews and demographics were collected with self-reported questionnaires. Body Mass Index (BMI) was calculated from height and weight collected at the baseline clinic visit.

### Statistical analyses

Participants demographics, breast cancer characteristics and treatment, MVPA, as well as self-reported and objective cognition measures (primary and secondary outcomes) were summarized in descriptive statistics; continuous variables are presented as mean (standard deviation, SD) and categorical variables were presented as number (%).

Multiple linear regression models (MLR) were fit to investigate associations between daily MVPA minutes and the two primary outcomes (processing speed and self-reported cognition), controlling for covariates: daily ActiGraph wear time, age, BMI, race (White vs. non-White), ethnicity (Hispanic vs. non-Hispanic), time since diagnosis (years), and cancer treatment. Due to the eligibility criteria of needing to be treated with chemotherapy and/or hormone therapy, we created a combined cancer treatment variable: “chemotherapy yes and hormone therapy yes” vs. “chemotherapy yes and hormone therapy no” vs. “chemotherapy no and hormone therapy yes.” NART was also included in the model as covariate for Processing Speed.

For secondary analyses assessing association between daily MVPA and the three cognitive domains, MLR was carried out with the same set of the covariates as primary analyses (including NART), with the exception of age since the domain scores, were already age-corrected during derivation. For the MLR for the primary outcomes and secondary analyses, the coefficient (β) of daily MVPA and its standard error (se) are presented with *p-*value indicating the significance of association. Residual plots were graphed to assess model fit.

For exploratory analyses assessing potential moderation of cancer treatment on relationship between daily minutes of MVPA and cognitive measures, two interaction terms, daily minutes of MVPA * years since diagnosis and daily minutes of MVPA * combined chemo-hormone treatment, were entered in the previous MLR models individually, such that each MLR only assessed one interaction at a time. Coefficients for the interaction term (β) and corresponding standard errors are presented with *p-*value indicating the significance of the modification.

Type I error (α-level) for all statistic tests was two-sided. Bonferroni correction was applied to the two primary outcomes to adjust for multiple comparisons by setting α = 0.025. No multiple comparison adjustment was made to secondary or exploratory analyses. All analyses were performed in R statistical program language (Team, 2020).

## Results

Of the 1,403 people who were screened for the study, 273 were initially eligible and attended the baseline visit. Of these, 253 were ultimately eligible, interested, and enrolled. The most frequent reasons for ineligibility were self-reporting too much physical activity 20.5% (*N* = 236), unable to or unsafe to increase physical activity 17% (*N* = 195), breast cancer diagnosed more than 5 years ago 13% (*N* = 149), no self-reported problems with cognition 12.3% (*N* = 141), or had not received chemotherapy or hormone therapy as part of their cancer treatments 8.3% (*N* = 96). All 253 participants completed all of the baseline measures including wearing the ActiGraph for the minimum required time.

Table 1 shows participant demographic and cancer treatment information. Participants were on average 58.5 (SD = 8.88) years old, ranging from 40 to 82 years old with an average BMI of 29.4 kg/m^2^ (SD = 6.17), ranging from 17.6 to 49.1 kg/m^2^. The majority of the participants identified as White (76.7%) and Non-Hispanic/non-Latina (83.8%). More than half reported currently working part or full time (58.9%) and 68.4% had a college or graduate degree. On average, participants were diagnosed 3 years prior to enrollment with 42.3% diagnosed with Stage 1, 43.5% with Stage 2, and 14.2% with Stage 3 breast cancer. A little more than half had been treated with chemotherapy, with 46.9% receiving neoadjuvant chemotherapy, 49.7% adjuvant chemotherapy, and 3.4% treated with both neoadjuvant and adjuvant chemotherapy. At baseline, 80.2% were receiving hormone therapy. One hundred and twelve (44.3%) were treated with both chemotherapy and hormone therapy, 108 (42.7%) were treated with hormone therapy without chemotherapy, and 33 (13.0%) were treated with chemotherapy without hormone therapy.

Participants wore the ActiGraph accelerometer for an average 7.2 days (SD = 1.41). Average daily MVPA was 16.5 min/day (SD = 16.48), ranging from 0.1 min/day to 117.3 min/day, with a 1^st^ and 3^rd^ quartile of 5.4 min/day and 21.4 min/day. Participants’ self-reported Cognitive Abilities was a mean T-score of 43.5 (SD = 7.28), ranging from 20.1 to 68.9, with a 1^st^ and 3^rd^ quartile of 39.4 and 47.6. Table 2 shows the means and standard deviations of each individual cognitive test and the composite cognitive domain z-scores.

For the primary self-report outcome, greater daily minutes of MVPA were significantly associated with better ratings on the PROMIS Cognitive Abilities measure (β = 0.070, se = 0.028, *p* = 0.012). This indicates that each additional 30 min of MVPA per day was associated with a 2.1 points higher rating of self-reported cognitive abilities. For the primary objective outcome, there was no significant association between daily minutes of MVPA and Processing Speed (β = 0.016, se = 0.051, *p* = 0.758). For the secondary outcomes, there was no significant association between daily minutes of MVPA and any of the three objective cognitive domains: Memory, Executive Function, and Attention Domains (See Table 3). Diagnostic plots indicated that the models fit the data adequately, there were neither notable violations of modeling assumptions nor influential outliers.

**FIGURE 1:**
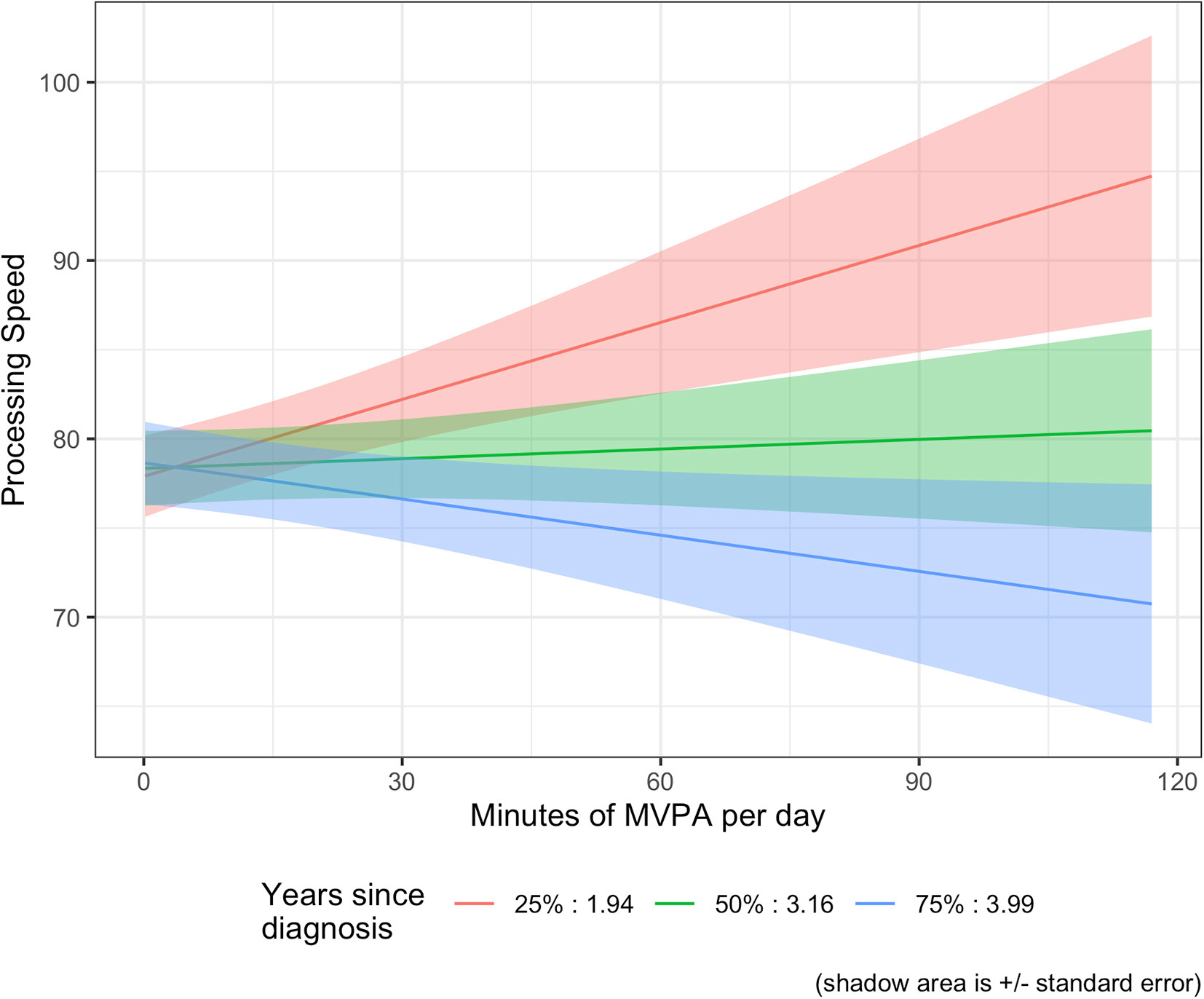
MVPA moderator analysis: processing speed and time since diagnosis (years). Graph of the estimate with time since diagnosis set at the 1^st^, 2^nd^, and 3^rd^ quartile of the data.

For the exploratory moderator analysis, we tested time since diagnosis (years) and treatment type (chemotherapy and/or hormone therapy) as potential moderators between daily minutes of MVPA and cognition. For self-reported cognitive abilities, neither time since diagnosis nor treatment with chemotherapy and/or hormone therapy were significant moderators (ps > 0.05). However, for objectively measured Processing Speed, there was a significant interaction for daily minutes of MVPA and years since diagnosis (β = −0.103, se = 0.043, *p* = 0.017). The results suggest that participants who were closer in time to their diagnosis had higher scores on the Digit Symbol Test with increasing minutes of MVPA, whereas people further from diagnosis had stable or slightly worsening scores with increasing minutes of MVPA (See Figure 1). For example, for participants who are 2 years post diagnosis, each additional 30 min per day of MVPA is associated with a 4.14 higher Processing Speed score. In contrast, for participants who are 4 years post diagnosis, each additional 30 min per day of MVPA is associated with a 2.04 lower Processing Speed score. There were no significant interactions for any of the other three secondary cognitive domains of interest for daily minutes of MVPA and years since diagnosis or treatment with chemotherapy (See Supplementary Table 1).

## Discussion

Consistent with our primary hypothesis and previous research, better self-reported cognition was associated with greater minutes of physical activity (Campbell et al., 2020; Brunet and Sharma, 2023; Jesús et al., 2023). This relationship is important as many women decrease their activity levels during and after breast cancer treatment (Howard-Anderson et al., 2012) and continue to have lower levels of PA than similar women without cancer (Tai et al., 2012; National Cancer Institute, 2019). The reductions in activity level may contribute to breast cancer survivors problems with cognition. Furthermore, these results support intervention trials that suggest increasing physical activity may be a potential way to improve self-reported cognition (Campbell et al., 2018; Hartman et al., 2018; Koevoets et al., 2022; Hiensch et al., 2023; Bender et al., 2024).

Contrary to our hypotheses, there were no significant relationships between any of the four objectively measured cognition domains (processing speed, cognitive memory, executive function, and attention domains) and daily minutes of MVPA. Although there was no significant main effect for processing speed, time since diagnosis was a significant moderator. Results suggest that only participants closer in time to their diagnosis had better processing speed with greater minutes of MPVA. This is consistent with a previous intervention trial that found improvements in processing speed for breast cancer survivors <2 years from diagnosis but not for those further from diagnosis (Hartman et al., 2018). One possible reason for this is cancer treatments’ acute effect on processing speed, with those who are more active during this time being the most protected. The association of exercise and processing speed may be more robust shortly after treatment when people are recovering from acute treatment effects. Although time since diagnosis was a significant moderator for processing speed, it was not a moderator of self-reported cognition. Time since diagnosis has not been widely examined as a moderator of the relationship between self-reported cognition and physical activity (Koevoets et al., 2022). Self-reported cognitive problems may represent more chronic and long-term symptoms that are not related to acute effects of treatments. More research is needed to understand the importance of timing of physical activity for different aspects of cognition.

The relationship between cognition and MVPA did not vary based on having received chemotherapy, hormone therapy, or both. This is consistent with an individual participant data meta-analysis by Hiensch et al. (2023) that indicated that treatment type was not a moderator of the effects of exercise on functioning (Hiensch et al., 2023). Similarly, a recently completed intervention trial with breast cancer survivors receiving endocrine therapy found that receiving chemotherapy did not moderate the results (Bender et al., 2024). These are important findings as much of the earlier research on cognition had focused on chemotherapy; however, our results and those of others suggest that regardless of treatment greater physical activity associated with better cognition.

Consistent with previous research, the only objectively assessed cognitive domain that was related to physical activity was processing speed (Campbell et al., 2018; Hartman et al., 2018; Bender et al., 2024). Measures of processing speed may be more sensitive to capturing cancer-related cognitive decline among breast cancer survivors (Wefel et al., 2004; Correa and Ahles, 2008; Ahles et al., 2010). Furthermore, the traditional neuropsychological tests used to assess cognition may not be able to capture the sometimes more subtle problems cancer survivors experience (Horowitz et al., 2018). As seen this this study, although participants had to self-report cognitive problems to be eligible, the average scores on the objective tests indicated the sample overall scored above average. Neuropsychological tests were originally designed to capture impairments in individuals with overt and severe brain pathologies including traumatic brain injuries, schizophrenia, and strokes (Gur et al., 2010; Barch et al., 2023). Although there is evidence of fMRI neurobiological basis for CRCD (Apple et al., 2017; Chen et al., 2017; Feng et al., 2020), the cognitive impact and detected changes on neuropsychological tests have been small (Ahles and Root, 2018). Utilization of cognitive neuroscience approaches to assessing cognition may be needed to understand the association between physical activity and other aspects of cognition (Horowitz et al., 2018; Lomeli et al., 2021).

While several of the analyses were exploratory and hypothesis generating in nature, the rigorous measurements of this study using an objective measure of physical activity and both self-reported and objective measures of cognition provide important contributions to the literature. The different results for objective and self-report cognition found in the current study are consistent with previous research (Koevoets et al., 2022; Brunet and Sharma, 2023; Tometich et al., 2023) and support the need for measuring both (Pullens et al., 2010; Hutchinson et al., 2012). Self-report is important in that it reflects the concerns, distress, and experience of the person. A study by Lycke et al. demonstrated that self-reported cognition, but not objective measures of cognition, was prospectively associated with overall quality of life (Lycke et al., 2019). Objective measures, on the other hand, can provide more detailed feedback on the specific areas where a person is experiencing cognitive decline, although these measures may not be sensitive to more subtle issues cancer survivors often experience. Despite the importance of both self-reported and objective cognition, a recent review of the literature on the effects of exercise on breast cancer-related cognitive impairment reported that none of the observational studies included both measures of cognition (Jesús et al., 2023). And a review of exercise and cancer-related cognition across cancer types found only 21% of articles presented both outcomes (Brunet and Sharma, 2023). Particularly as the existing research on physical activity and CRCD remains inconclusive (Brunet and Sharma, 2023), understanding the relationship of physical activity with all aspects of cognition is important for supporting quality of life in cancer survivors.

### Limitations

Several limitations of the study should also be noted including the cross-sectional design prevents testing for causal relationships. As this is baseline data from an intervention trial, we will have data to test causality and examine if changing minutes of MVPA can improve cognition. Furthermore, future studies utilizing compositional data analysis considering the entire 24-h day and multiple behaviors including sleep, sedentary behavior, light physical activity, and moderate-vigorous physical activities may provide more robust statistical analyses (Verswijveren et al., 2022). The sample was predominantly white, non-Hispanic, and well educated limiting the generalizability of the results. The eligibility criteria required participants to have completed treatment at least 6 months prior to enrollment. Exercise prior to diagnosis, during treatment, and shortly after treatment may all play important roles in preserving or improving cognition that could not be assessed in our study.

Eligibility also included self-report engaging in <60 min of MVPA, in 10-min bouts. Since eligibility was based on self-report, some participants had very high levels of MVPA from the ActiGraph that counted any minute of MVPA. Even so, diagnostic tests and residual plots indicated that there were no unduly influential outliers, and the models fit the data well. One possible reason that some participants had more activity on the ActiGraph than self-reported is that the self-reported screener question likely led people to report leisure time physical activity and may have missed work-related activity. A limitation of objective measures of physical activity, such as the ActiGraph, is that it does not provide any information on the types of activity a person engaged in. Notably, leisure-time physical activity has greater benefits for cardiovascular disease (Cillekens et al., 2022; Edimo Dikobo et al., 2023) and dementia (Rasmussen et al., 2022) than does work-related physical activity. It is possible that leisure-time physical activity may be more important for improving cancer-related cognition than work-related physical activity. To provide the best recommendations for improving cognition, understanding if there are differential benefits based on type of activity will be important for future research. Additionally, the lack of significant relationship between objectively measured cognition and MVPA may be related to the type of activity participants were engaged in at baseline. It may also be due to the type of traditional neuropsychological tests used. Future research utilizing brain imaging and more sensitive measures are needed to understand the relationship of physical activity with all aspects of cognition (Horowitz et al., 2018; Lomeli et al., 2021). There absence of individual baseline for cognitive abilities and physical activity levels pre-cancer diagnosis may introduce high variability and potentially contributing to a negative impact on our statistical findings. Finally, participants had to self-report poor cognition and low physical activity to be eligible for the study. Although, there was sufficient variability in the measures for the current analyses, not enrolling participants across the spectrum of possible self-reported cognition and activity level may have limited our ability to detect significant relationships between cognition and activity.

In summary, results of the current study provide support physical activity may be related to self-reported cognition and objective processing speed for those closer to their time since diagnosis. This supports the testing of physical activity as potential intervention to determine if it can improve cognition in cancer survivors. Future research aimed at teasing apart who benefits from exercise, what types of exercise, and when the exercise needs to occur for the benefits will aid in the development of personalized recommendation to support cognitive health in cancer survivors.

## Supplementary Material

Supplemental Table

## Figures and Tables

**TABLE 1:** Demographic and cancer treatment characteristics (*N* = 253).

**Age, years, mean (SD)**	58.5 (8.88)
**BMI, *kg/m*^2^, mean (SD)**	29.4 (6.17)
**NART, mean (SD)**	38.5 (9.26)
**Education, *n* (%)**	
Some college or less	80 (31.6)
College graduate	107 (42.3)
Graduate degree	66 (26.1)
**Marital status, *n* (%)**	
Divorced/separated/widowed	69 (27.2)
Living with partner	172 (68.0)
Never married	12 (4.7)
**Ethnicity *n* (%)**	
Hispanic/latina	41 (16.2)
Non-hispanic/latina	212 (83.8)
**Race *n* (%)**	
American Indian or Alaska Native	3 (1.2)
Asian	27 (10.7)
Black/African American	8 (3.2)
Native Hawaiian or Other Pacific Islander	1 (0.4)
White	194 (76.7)
More than one race/other	20 (7.9)
**Cancer stage, *n* (%)**	
Stage 1	107 (42.3)
Stage 2	110 (43.5)
Stage 3	36 (14.2)
**Cancer hormone receptor status, *n* (%)**	
ER+, HER2−	184 (72.7)
ER+, HER2+	36 (14.2)
ER−, HER2+	10 (4.0)
ER−, HER2−	23 (9.1)
**Hormone therapy, *n* (%)**	
Currently taking	203 (80.2)
Previously took	17 (6.7)
Not prescribed	33 (13.0)
**Received chemotherapy, *n* (%)**	145 (57.3)
Neoadjuvant	68 (46.9)
Adjuvant	72 (49.7)
Both	5 (3.4)
**Combined chemotherapy and hormone therapy, *n* (%)**	
Chemotherapy yes and hormone therapy yes	112 (44.3)
Chemotherapy no and hormone therapy yes	108 (42.7)
Chemotherapy yes and hormone therapy no	33 (13.0)
**Time since diagnosis, years, mean (SD)**	3.0 (1.27)
**Received radiation, *n* (%)**	206 (81.4)
**ActiGraph minutes, mean (SD)**	
Daily minutes of MVPA	16.5 (16.48)
Daily minutes of wear time	864.5 (60.76)

**TABLE 2:** Cognitive tests (*N* = 253).

	Mean, (SD)
**Self-reported cognitive abilities T-score**	43.5 (7.28)
**Processing speed - digit symbol score**	77.1 (14.00)
**Memory domain z-score**	0.000 (0.73)
HVLT total immediate recall score	23.5 (5.01)
HVLT delayed recall score	7.4 (3.23)
NIH toolbox list sorting working memory age-corrected standard score	105.4 (14.23)
NIH toolbox picture sequence memory age-corrected standard score	107.3 (17.05)
**Executive function domain z-score**	−0.001 (0.79)
Trail making test B (seconds)	67.6 (30.02)
NIH toolbox dimensional change card sort test age-corrected standard score	106.4 (16.64)
**Attention domain z-score**	0.004 (0.50)
NIH toolbox flanker inhibitory control and attention test age-corrected standard score	89.7 (11.06)
CPT detectability (d’) T-score	45.2 (8.74)
CPT variability T-score	45.9 (6.78)
CPT hit reaction time block change T-score	49.7 (8.37)
CPT hit reaction time inter-stimulus intervals change T-score	54.4 (9.49)

**TABLE 3:** Linear effects models of MVPA with cognition, controlling for covariates (*N* = 253).

	Estimate	Std Error	*p* value
PROMIS Cognitive Abilities	0.070	0.028	0.012*
Processing Speed	0.016	0.051	0.758
Memory Domain	0.001	0.003	0.794
Executive Function Domain	0.003	0.003	0.337
Attention Domain	0.002	0.002	0.222

* *p* < 0.05.

Covariates controlled for: daily ActiGraph wear time, BMI, race (White vs. non-White), ethnicity (Hispanic vs. non-Hispanic), time since diagnosis, cancer treatment (chemo and hormone yes, chemo yes and hormone no, chemo no, and hormone yes). Age was controlled for in all models except for those with the Domain scores where age was corrected for during derivation. NART was controlled for in models with objective cognitive measures.

## Data Availability

The raw data supporting the conclusions of this article will be made available by the authors, without undue reservation.
