# Supplementary material for: Relationship of physical activity and cognitive functioning among breast cancer survivors: a cross-sectional analysis
Source: Front Cognit. 2024 May 9;3:1332960. doi: 10.3389/fcogn.2024.1332960 (PMC11526472; doi:10.3389/fcogn.2024.1332960)
Supplement: Supplementary file 1 [file Table_1.docx]

| Supplemental Table 1: Moderator Analyses | | | |
| --- | --- | --- | --- |
| **Time Since Diagnosis (years) Moderator Analysis** | **Estimate** | **Std. Error** | **P-value** |
| **Self-Report Cognitive Abilities** |  |  |  |
| Daily min of MVPA (Ref: time since dx =0) | 0.049 | 0.080 | 0.539 |
| Daily min of MVPA * Time since diagnosis | 0.007 | 0.024 | 0.777 |
| **Processing Speed** |  |  |  |
| Daily min of MVPA (Ref: time since dx =0) | 0.344 | 0.146 | 0.019 |
| Daily min of MVPA * Time since diagnosis | -0.103 | 0.043 | 0.017 |
| **Memory Domain** |  |  |  |
| Daily min of MVPA (Ref: time since dx =0) | 0.007 | 0.008 | 0.351 |
| Daily min of MVPA * Time since diagnosis | -0.002 | 0.002 | 0.369 |
| **Executive Functioning Domain** |  |  |  |
| Daily min of MVPA (Ref: time since dx =0) | 0.006 | 0.008 | 0.501 |
| Daily min of MVPA * Time since diagnosis | -0.001 | 0.003 | 0.718 |
| **Attention Domain** |  |  |  |
| Daily min of MVPA (Ref: time since dx =0) | 0.001 | 0.005 | 0.926 |
| Daily min of MVPA * Time since diagnosis | 0.001 | 0.002 | 0.718 |
|  |  |  |  |
| **Cancer Treatment Moderator Analysis** | **Estimate** | **Std. Error** | **P-value** |
| **Self-Report Cognitive Abilities** |  |  |  |
| Daily min of MVPA (Ref group: Hormone Yes, Chemo No) | 0.092 | 0.041 | 0.028 |
| Daily min of MVPA * Hormone Yes, Chemo Yes | -0.027 | 0.056 | 0.636 |
| Daily min of MVPA * Hormone No, Chemo Yes | -0.097 | 0.102 | 0.345 |
| **Processing Speed** |  |  |  |
| Daily min of MVPA (Ref group: Hormone Yes, Chemo No) | 0.031 | 0.076 | 0.685 |
| Daily min of MVPA * Hormone Yes, Chemo Yes | -0.026 | 0.104 | 0.803 |
| Daily min of MVPA * Hormone No, Chemo Yes | -0.034 | 0.189 | 0.858 |
| **Memory Domain** |  |  |  |
| Daily min of MVPA (Ref group: Hormone Yes, Chemo No) | 0.000 | 0.004 | 0.967 |
| Daily min of MVPA * Hormone Yes, Chemo Yes | 0.003 | 0.006 | 0.594 |
| Daily min of MVPA * Hormone No, Chemo Yes | -0.006 | 0.010 | 0.534 |
| **Executive Functioning Domain** |  |  |  |
| Daily min of MVPA (Ref group: Hormone Yes, Chemo No) | 0.007 | 0.004 | 0.096 |
| Daily min of MVPA * Hormone Yes, Chemo Yes | -0.009 | 0.006 | 0.138 |
| Daily min of MVPA * Hormone No, Chemo Yes | -0.003 | 0.011 | 0.797 |
| **Attention Domain** |  |  |  |
| Daily min of MVPA (Ref group: Hormone Yes, Chemo No) | 0.002 | 0.003 | 0.510 |
| Daily min of MVPA * Hormone Yes, Chemo Yes | -0.0002 | 0.004 | 0.958 |
| Daily min of MVPA * Hormone No, Chemo Yes | 0.006 | 0.007 | 0.363 |
